# Supplementary material for: Adiponectin pathway activation dampens inflammation and enhances alveolar macrophage fungal killing via LC3-associated phagocytosis
Source: bioRxiv. 2024 Jun 26:2024.06.24.600373. Preprint. [Version 1] doi: 10.1101/2024.06.24.600373 (PMC11230297; doi:10.1101/2024.06.24.600373)
Supplement: Supplementary file 1 [file NIHPP2024.06.24.600373v1-supplement-1.pdf]

bioRxiv preprint doi: https://doi.org/10.1101/2024.06.24.600373; this version posted June 26, 2024. The copyright holder for this preprint (which was not certified by peer review) is the author/funder, who has granted bioRxiv a license to display the preprint in perpetuity. It is made available under aCC-BY 4.0 International license.

Supplemental Information

Supplemental Methods

Mice

An additional strain of Adipoq-/- mice was obtained from Dr. Philipp Scherer (University of Texas-Southwestern) and used for survival, fungal burden, histology, flow cytometric analysis of BALF cells, qRT-PCR of selected inflammatory and *Adipor* genes, and ELISA for TNF as described in the Materials and Methods section.

Quantification of AM AdipoR1 expression using flow cytometry

After ex-vivo AM extraction and culture as mentioned in the methods, AMs were fixed using IC fixation buffer. Fc Block was used to eliminate non-specific Fc-mediated interactions. The cells were then stained at a 1:100 dilution with primary antibody with Adiponectin Receptor 1 Recombinant Rabbit Monoclonal Antibody (SC69-04) followed by staining with Alexa Fluor 488-conjugated goat anti rabbit IgG as the secondary antibody. All the unstained and only primary antibody staining controls were included.

Quantification of AM *Adipor1* and *Adipor2* receptor gene expression

RNA extraction from AMs was done using Qiagen RNeasy Mini kit, following the manufacturer's protocol. Quantitative RT-PCR was performed with 20ng of cDNA using AdipoR1 and AdipoR2 forward and reverse primers ordered from IDT Integrated Technologies. Gene Expression Master Mix (ThermoFisher Scientific) was used, with β-actin used for signal normalization.

RNA sequencing and analysis

Sample Preparation

AMs were isolated from APN-/- mice, infected/uninfected for 10 hours (1:9 cells/conidia) and AdipoRon/vehicle treated 24 hours before infection, with cells lysed and RNA isolated as described in Materials and Methods.

Library preparation and sequencing

Total RNA samples were first evaluated for their quantity and quality using Agilent TapeStation. All

bioRxiv preprint doi: https://doi.org/10.1101/2024.06.24.600373; this version posted June 26, 2024. The copyright holder for this preprint (which was not certified by peer review) is the author/funder, who has granted bioRxiv a license to display the preprint in perpetuity. It is made available under aCC-BY 4.0 International license.

the samples were good quality with RIN (RNA Integrity Number) of 9.6-10. One hundred nanograms of total RNA was used for library preparation with the Illumina Stranded mRNA Prep, Ligation kit (lllumina), following the manufacturer's instruction. Each resulting uniquely dual-indexed library was quantified and quality accessed by Qubit and Agilent TapeStation, and multiple libraries were pooled in equal molarity. The pooled libraries were sequenced with 2×150bp paired-end configuration on an Illumina NovaSeq X PLUS sequencer.

RNA-seq data analysis

The sequencing reads were first quality checked using FastQC (v.0.11.5, Babraham Bioinformatics, Cambridge, UK) for quality control. The sequence data were then mapped to the mouse reference genome mm10 using the RNA-seq aligner STAR (v.2.7.10a)[52] with the following parameter: "--outSAMmapqUnique 60". To evaluate quality of the RNA-seq data, the number of reads that fell into different annotated regions (exonic, intronic, splicing junction, intergenic, promoter, UTR, etc.) of the reference genome was assessed using bamutils (from ngsutils v.0.4.17).[53] Uniquely mapped reads were used to quantify the gene level expression employing featureCounts (subread v.2.0.3)[54] with the following parameters: "-s 2 -Q 10". The data was normalized using TMM (trimmed mean of M values) method. Differential expression analysis was performed using edgeR (v.4.0.1).[54,55] False discovery rate (FDR) was computed from p-values using the Benjamini-Hochberg procedure. Gene Ontology (GO) pathway enrichment analyses were performed with the R package clusterProfiler.[56,57]

bioRxiv preprint doi: https://doi.org/10.1101/2024.06.24.600373; this version posted June 26, 2024. The copyright holder for this preprint (which was not certified by peer review) is the author/funder, who has granted bioRxiv a license to display the preprint in perpetuity. It is made available under aCC-BY 4.0 International license.

Supplemental Figure S1.

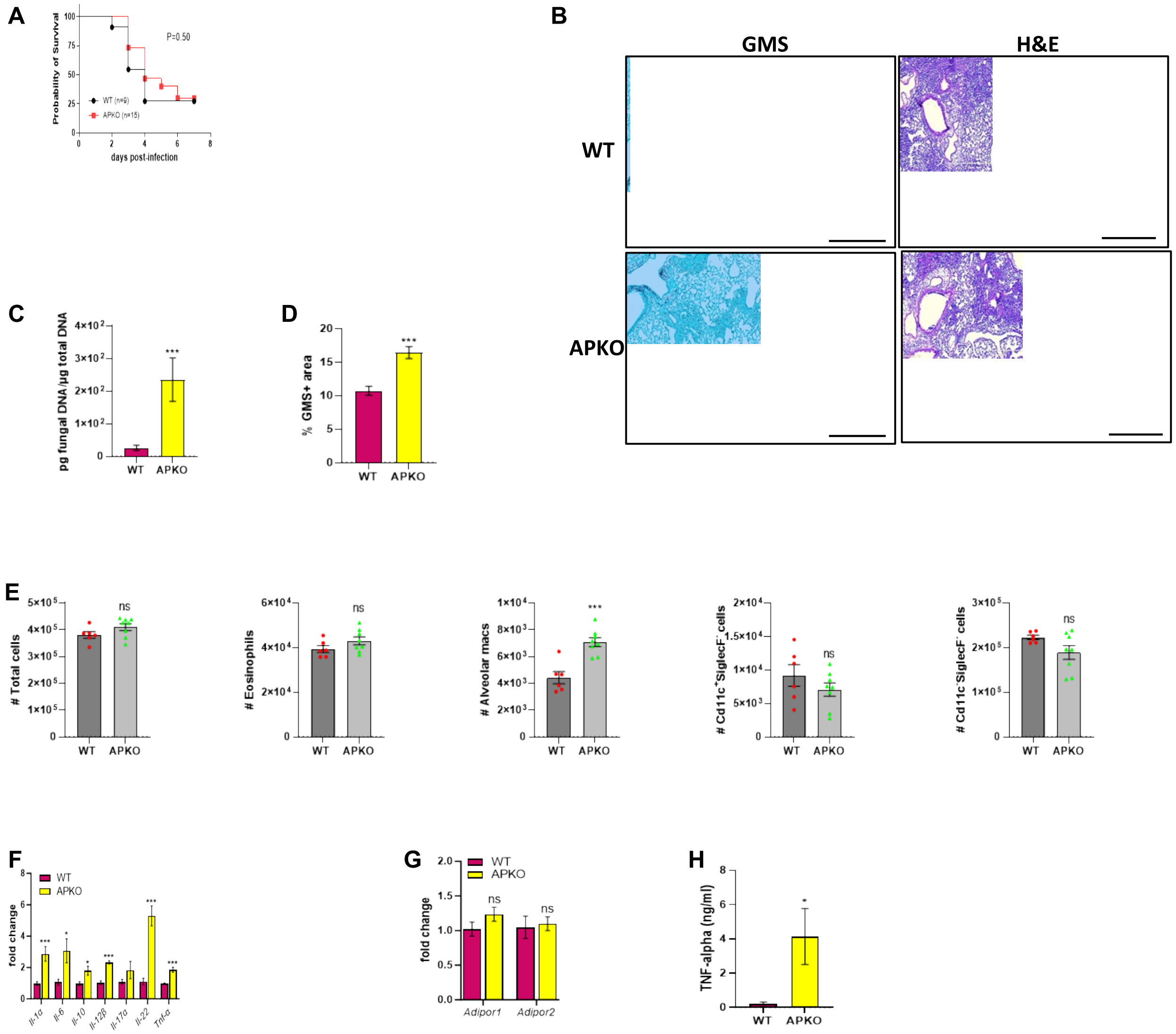

bioRxiv preprint doi: https://doi.org/10.1101/2024.06.24.600373; this version posted June 26, 2024. The copyright holder for this preprint (which was not certified by peer review) is the author/funder, who has granted bioRxiv a license to display the preprint in perpetuity. It is made available under aCC-BY 4.0 International license.

Fig. S1. Inflammatory phenotype of APN-/- mice from a second strain.

Wild-type (C57BL/6) and APKO (Second *Adipoq*$^{-/-}$ mice from Scherer's laboratory) were neutrophil depleted and involuntarily aspirated 1 – 1.5 × 10^{7} of conidia as described in Materials and Methods.

A. Survival rate. *N* = 9 and 15 mice per group, respectively.
B. Representative GMS and H&E lung sections.
C. Fungal burden determined by quantitative PCR of fungal DNA from lung homogenates.
D. Fungal burden determined by quantification of GMS staining.
E. Total number of CD45^{+} cells, eosinophils (CD45^{+}Ly6G^{-}CD11c^{-}SiglecF^{+}), AMs (CD45^{+}Ly6G^{-}CD11c^{+}SiglecF^{+}), CD11c^{+}SiglecF^{-} (CD45^{+}Ly6G^{-} CD11c^{+}SiglecF^{-}), and CD11c^{-}SiglecF^{-} (CD45^{+}Ly6G^{-}CD11c^{-}SiglecF^{-}) cells isolated from the mice with IA as determined by flow cytometry. *N* = 4-6 mice per group.
F. qRT-PCR analysis for mRNA expression of the indicated cytokines.
G. qRT-PCR analysis for mRNA expression of *Adipor1* and *Adipor2*.
H. TNFα secretion in BALF quantified at the protein level by ELISA.

Data are a summary of two to three independently performed experiments. *$p < 0.05$, **$p < 0.01$, ***$p < 0.001$.

bioRxiv preprint doi: https://doi.org/10.1101/2024.06.24.600373; this version posted June 26, 2024. The copyright holder for this preprint (which was not certified by peer review) is the author/funder, who has granted bioRxiv a license to display the preprint in perpetuity. It is made available under aCC-BY 4.0 International license.

Supplemental Figure S2.

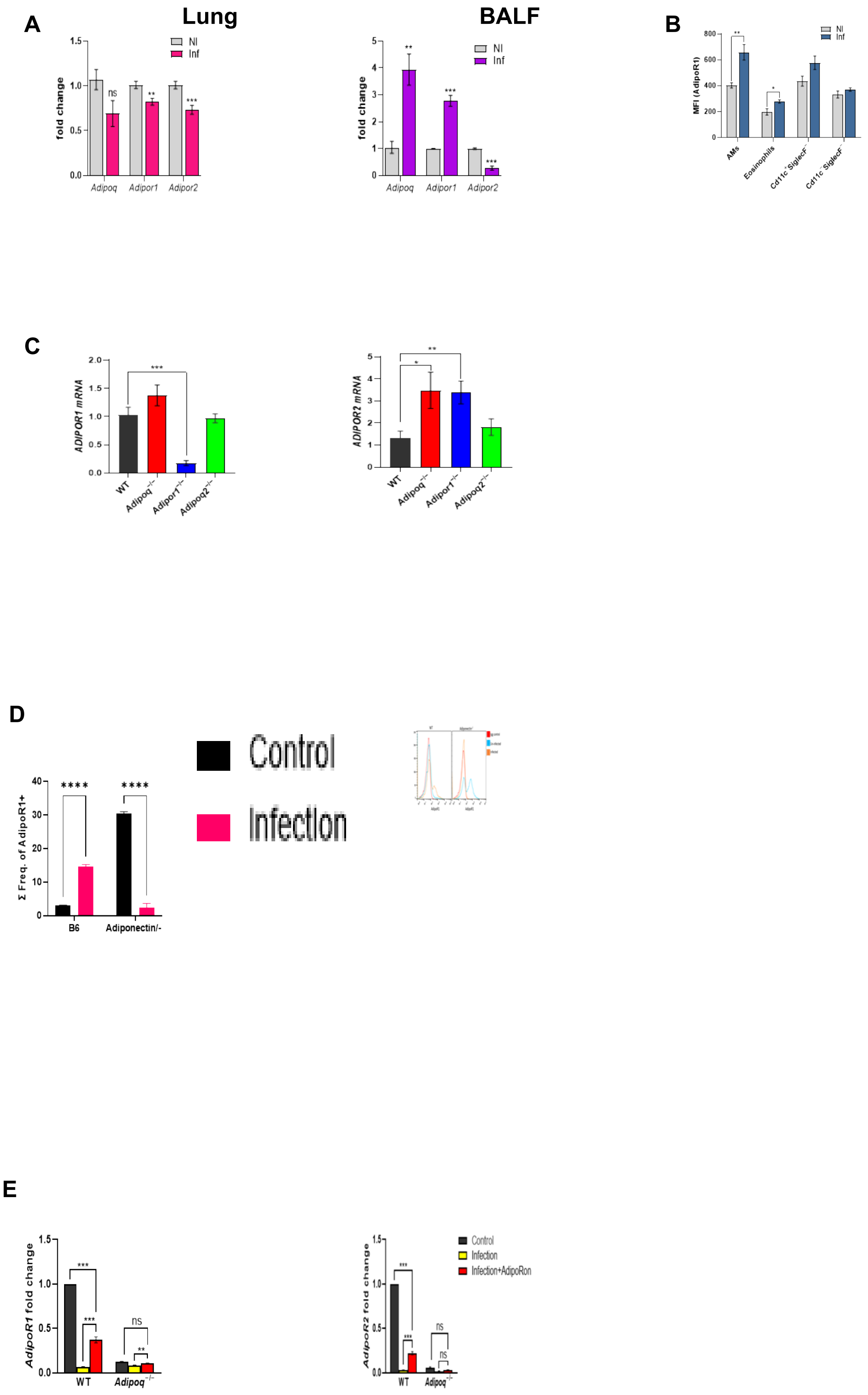

bioRxiv preprint doi: https://doi.org/10.1101/2024.06.24.600373; this version posted June 26, 2024. The copyright holder for this preprint (which was not certified by peer review) is the author/funder, who has granted bioRxiv a license to display the preprint in perpetuity. It is made available under aCC-BY 4.0 International license.

Fig. S2. AdipoRs expression in the Lung and BALF in APN pathway-deficient mice with invasive aspergillosis.

A. Expression of *Adipoq* and *Adipor* genes in lung homogenates and BALF cells from non-infected (NI) and conidia-infected WT mice. Lung and BALF were collected from the mice at 3 dpi.
B. Summary of median fluorescence intensities of AdipoR1 staining on AMs (CD45^{+}Ly6G^{-}CD11c^{+}SiglecF^{+}), eosinophils (CD45^{+}Ly6G^{-}CD11c^{-}SiglecF^{+}), CD11c^{+}SiglecF^{-} (CD45^{+}Ly6G^{-} CD11c^{+}SiglecF^{-}), and CD11c^{-}SiglecF^{-} (CD45^{+}Ly6G^{-}CD11c^{-}SiglecF^{-}) cells from non-infected (NI) and conidia-infected WT mice.
C. qRT-PCR analysis for mRNA expression of *Adipor1* and *Adipor2* in lung homogenates. Wild-type (C57BL/6), *Adipoq*$^{-/-}$, *AdipoR1*$^{-/-}$, and *AdipoR2*$^{-/-}$mice were neutrophil depleted and involuntarily aspirated *A. fumigatus* conidia.
D. Flow cytometry staining of AdipoR1. Frequency of AdipoR1+ in ex-vivo cultured AMs in WT and *Adipoq*$^{-/-}$ mice. The histogram represents the AdipoR1 peak relative to IgG control in infected vs uninfected.
E. qRT-PCR analysis for mRNA expression of *Adipor1* and *Adipor2* from the ex-vivo cultured AMs. The AMs were challenged with AF-293 conidia with or without AdipoRon treatment.

Data are a summary of two to three independently performed experiments. *$p < 0.05$, **$p < 0.01$, ***$p < 0.001$.

bioRxiv preprint doi: https://doi.org/10.1101/2024.06.24.600373; this version posted June 26, 2024. The copyright holder for this preprint (which was not certified by peer review) is the author/funder, who has granted bioRxiv a license to display the preprint in perpetuity. It is made available under aCC-BY 4.0 International license.

Supplemental Figure S3.

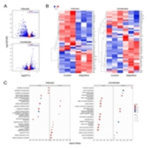

779

bioRxiv preprint doi: https://doi.org/10.1101/2024.06.24.600373; this version posted June 26, 2024. The copyright holder for this preprint (which was not certified by peer review) is the author/funder, who has granted bioRxiv a license to display the preprint in perpetuity. It is made available under aCC-BY 4.0 International license.

Fig. S3 Gene expression in AdipoRon treated/untreated and infected/ uninfected *Adipoq*$^{-/-}$ AMs by RNAseq analysis.

AMs were infected with swollen AF293 conidia with 1:9 cells/conidia for 10 hours, or left uninfected, with or without AdipoRon treatment, followed by RNA extraction for RNAseq analysis (N=3/group).

A. Volcano plot depicting relative changes in gene expression of AdipoRon-treatment in APN-/- AMS, infected (top) or uninfected (bottom).
B. Heat map representation of the genes with highest differential expression in infected (left) and uninfected (right) APN-/- AMs.
C. GSEA-GO analysis of gene pathways that are both differentially expressed by AdipoRon treatment in infected (left) and uninfected (right) APN-/- AMs.

bioRxiv preprint doi: https://doi.org/10.1101/2024.06.24.600373; this version posted June 26, 2024. The copyright holder for this preprint (which was not certified by peer review) is the author/funder, who has granted bioRxiv a license to display the preprint in perpetuity. It is made available under aCC-BY 4.0 International license.

Supplemental Video SV1. Time course of uptake and killing in WT and APN-/- AMs.

Alveolar macrophages were plated and infected as mentioned in the methods. Cells were live imaged by confocal microscopy for T= 8min under 40X water immersion lens. The black arrows indicate the live conidia at T=0 min and the white arrows indicate the conidia at T=8min.
